# 

**Published:** 1895-12-14

**Authors:** 


**Notices of Births, Deaths, and Marriages** are inserted in this column at a charge of 2s. 6d. for an announcement not exceeding four lines.

**Notice to Advertisers and Subscribers.**

All Advertisements, Orders for Copies of THE HOSPITAL and Business Communications should be addressed to **THE MANAGER, (Not to The Editor)**, THE HOSPITAL, 428, Strand, London, W.C.

The Cost of Subscription, post free, is as follows:—Per week, 2½d.; per quarter, 2s. 8d.; per half-year, 5s. 3d.; per annum, 10s. 6d. Foreign:—Per Annum, 15s. 2d.; payable, in all cases, in advance.

### NOTICE TO CORRESPONDENTS.

All MS., Letters, Books for Review, and other matters intended for the Editor should be addressed THE EDITOR, THE LODGE, PORCHESTER SQUARE, LONDON, W.

The Editor cannot undertake to return rejected MS., even when accompanied by stamped directed envelope.

**"Burdett's Hospital Annual," 1895,**

Sixth year of publication. 950 pp. Scarlet cloth, gilt lettered. Price 5s. A most complete guide to British, Colonial, Indian, and American Hospitals, Dispensaries, Nursing and Convalescent Institutions, and Asylums. A few complete sets of the preceding five issues of the "Annual" may still be obtained on application to the Publishers, THE SCIENTIFIC PRESS (Limited), 428, Strand, London, W.C.

**NOTICE.—Vol. XVIII. of "The Hospital" (April to September, 1895), in handsome cloth binding, is now on sale at the Office of this Journal. Price 7s. 6d. Cases for binding the Numbers contained in this Volume, 1s. 6d. Index to Vol. XVIII., 2½d., post free.**

**The Monthly Part for December is now ready, price 6d., by post, 8d.**

**IMPORTANT NOTICE.**

**On account of the Christmas Holidays, we beg to announce that we shall go to press with our issue of the 28th INSTANT on MONDAY NIGHT, the 23rd INST. Advertisements intended for insertion in this issue, should therefore reach us BY NOON ON MONDAY, the 23rd inst.**

### Scraps and Gleanings.

At the annual meeting of subscribers to the Belfast Royal Hospital attention was called to the fact that there were two beds still unendowed in the consumptive department.

The Finance Committee of the Edinburgh Royal Infirmary have applied £1,886 out of the £2,627 surplus balance on the year's transactions of the institution to wiping off the remains of the old building fund debt.

The new medical hall in connection with Queen Margaret College, Glasgow, has been formally opened. The history of this college, and especially of its medical school, has been one of growing prosperity. It was organised in 1889 in consequence of a request made by several ladies that the Executive Council of the college should give them facilities to study medicine there. The Council was financially put in a position to acquiesce in this proposal through the generosity of Mrs. Elder.

The financial statement of the Leeds Ladies' Hospital Fund is not of a very satisfactory order, when compared with last year's figures, but this society is so indefatigable in its endeavours, that a better state of things may be looked for.

At the annual meeting of the subscribers to the Belfast Royal Hospital the board of management reported that the Throne Hospitals have been maintained in their usual efficiency. Four hundred and fifty-five patients have been treated in the convalescent department, 21 in the consumptive, and 116 in the children's hospital.

A ball was held last week at the Assembly Rooms, Hull, in aid of the Hull Hospital for Women and Orthopædics. This is the third venture of the kind, the one held last year resulting in a sum only just short of £100. The hospital is doing a good work, locally, and for an organisation of its kind a large one.

The Northallerton Cottage Hospital Committee give a good report of the working of the institution. The accounts, too, are fairly satisfactory, and the debt to the bank has been reduced.

Over 100 useful articles for the use of the patients of the Salford Royal Hospital have been received from the Manchester, South-East Lancashire, and East Cheshire Needlework Guild.

The Lord Chamberlain, by the desire of the Queen, has forwarded 104 lbs. of cast linen for the use of the Middlesex Hospital.

Lord Wolverton has sent a present of 50 rabbits for the benefit of the patients at the Metropolitan Hospital, Kingsland Road.

The treasurer of the Monmouth Hospital and Dispensary has received from Major Lister a cheque for £134, the balance of the fund presented to the Duke and Duchess of Beaufort by their Monmouthshire friends on the occasion of their golden wedding.

It is intended to hold a grand fête and bazaar at Ball's Bridge, Dublin, in May, 1896, in aid of the fund required to effect the amalgamation of St. Mark's Ophthalmic Hospital, Lincoln Place, and the National Eye and Ear Infirmary, Molesworth Street.

The number of pensioners at the Aged Pilgrims' Asylum, Hornsey Rise, N., is now 1,348, and the annual expenditure in pensions is £8,700. To meet this amount the ordinary income is inadequate. The pensioners are equally divided between the metropolis and other parts of the country.

The expenditure at the Brompton Consumption Hospital is necessarily about £15,000 a year beyond the more or less permanent income, and, considering this, it is a matter of much regret that the donations last year exhibited the considerable decrease of £2,293, and the year closed with a debit balance of no less than £2,803.

The Governor of the Punjab has ordered a test to be put upon criminals with a view to experimenting upon the effect of the consumption of water. His orders are that half the prisoners in gaols shall be kept on fresh water, and half on boiled, with a view to recording if the latter has any real effect in preventing sickness.

Already the Newcastle Infirmary funds are as much as £1,250 in advance of the total for the corresponding nine months in 1894. Every source of revenue of this institution has been more productive than usual, save that of general subscriptions, and even here the decrease of £82 does not constitute any alarming fluctuation.

In aid of the Birmingham and Midland Hospital for Women—an institution which unostentatiously has been alleviating much suffering in the Midlands for a considerable number of years—a sale of work was opened last week, in the out-patient department. The immediate object of the bazaar is to clear off a debt of £230 on the Hospital for In-Patients at Sparkhill, and also to relieve somewhat the anxiety of the committee in view of the fact that the expenditure last year was £560 in excess of the income.

We notice that the expeditionary forces to Ashanti have been ordered to be provided with 10 large portable Pasteur filters for the use of the troops. The publicity given lately to filtration of water is already bearing good fruits in this direction.

Her Majesty the Queen has commanded that 100 lbs. of cast linen be forwarded for the service of the Cancer Hospital, Brompton.

The annual sale of work held by the Ladies' Committee of the National Orthopædic Hospital on December 6th and 7th realised over £90.

Mr. Weston, a well-known inhabitant of Macclesfield, has given another £50 towards the Thornycroft Convalescent Fund, making in the aggregate £450 contributed by that gentleman.

The committee of the Industrial Exhibition held at Macclesfield have decided to hand over a sum equal to 5 per cent. of the gross takings to the children's ward of the General Infirmary.

The annual meeting of the Croydon General Hospital showed a very gratifying report. The record of work during the past year was stated to have been excellent. The number of patients under treatment in the wards shows a large increase over any former year. There appears, however, to be a lamentable falling off in legacies, and in the annual income, generally.

Over £12,000 has already been subscribed towards the establishment of a West of Scotland Consumptive Hospital.

Mr. Ogilvey Dalgleish, of Errol Park, has offered £3,500 towards the erection of a nurses' home in connection with the Dundee Royal Infirmary.

### THE STUDENT OF MEDICINE.

### APPOINTMENTS.

Mr. E. R. Badcock, appointed Assistant Medical Superintendent for the Infirmary of the Lewisham Union. R. C. Brown, M.B., B.C., B.A.Cantab., appointed Senior House Surgeon of the Blackburn and East Lancashire Infirmary. P. S. Buchanan, M.B., C.M.Glasg., appointed Additional Assistant Medical Officer to the Town Hospital, Glasgow. T. W. Cole, B.A.Dub., M.B., B.Ch., appointed Medical Officer of Health to the Bolsover District Council. F. Dale, M.D.Cantab., F.R.C.S.Eng., appointed Honorary Consulting Surgeon to the Scarborough Hospital and Dispensary. E. P. Dickin, M.B., C.M.Edin., M.R.C.S.Eng., L.R.C.P.Lond., appointed House Surgeon to the General Infirmary, Northampton. T. W. Elliott, appointed Medical Officer for the Tonbridge Union. Dr. Fulton, appointed Medical Officer of Health to the Stevenston Parish Council. J. W. Geddes, M.B., C.M.Edin., appointed Junior Assistant Medical Officer to the Durham County Asylum, Winterton, Ferryhill. W. C. Hamilton, M.B., C.M.Edin., appointed Senior House Surgeon of the South Devon and East Cornwall Hospital, Plymouth. W. E. Harker, M.D.McGill, L.R.C.P.I., appointed Medical Officer of Health to the Tyne Port Sanitary Authority. J. E. Hewlett, M.B., C.M. Edin., appointed Junior House Surgeon to the Blackburn and East Lancashire Infirmary. R. Jardine, M.D.Edin., M.R.C.S. Eng., F.F.P.S.Glasg., appointed Assistant Physician to the Glasgow Maternity Hospital. E. Lyons, B.A., M.B., B.Ch., B.A.O.T.C.D., L.M.(Rotunda), appointed Junior Resident Surgeon to the Jervis Street Hospital, Dublin. F. W. Mackay, appointed Ophthalmic Surgeon to the Western Dispensary, Edinburgh. C. J. Perrott, L.R.C.P., L.R.C.S.I., appointed Certifying Factory Surgeon for Kingswood, St. George, &c. Dr. Pridmore, appointed Medical Officer for the Weymouth District of the Weymouth Union. Dr. Pringle, appointed Medical Officer of

### THE STUDENT OF MEDICINE—*continued.*

Health for the No. 3 District of the Bridgwater Union. Dr. Reynolds, appointed Medical Officer for the Morchard Bishop District of the Crediton Union. J. A. Smith, M.R.C.S.Eng., L.S.A., appointed Medical Officer for the Flockton District of the Wakefield Union. J. S. Tew, M.D.Durh., M.B., M.R.C.S., appointed Medical Officer of Health for the Sevenoaks, Southborough, Tenterden, and Tonbridge Urban Sanitary Districts, and the Bromley, Cranbrook, Maidstone, Sevenoaks, Tenterden, and Tonbridge Rural Sanitary District. L. Turner, M.D., M.B., C.M., appointed Physician for Diseases of the Ear and Throat, Western Dispensary, Edinburgh. D. Walsh, M.B., C.M.Edin., late Assistant Physician, Western Skin Hospital, London, W., appointed Physician. C. Whitehall-Cooke, M.D.Lond., M.R.C.S., L.R.C.P., appointed Honorary Medical Officer to the Kilburn, Maida Vale, and St. John's Wood Dispensary. W. T. Williams, M.R.C.S.Eng., appointed Medical Officer for the Second District of the Township of Manchester. H. S. Byers, appointed House Surgeon to the Stockton and Thornaby Hospital.

#### VACANCIES.

The following vacancies are announced this week, the amount of salary in each case being given after the name of the institution:—

##### Resident Medical Officers.

Warneford Hospital, Leamington.—House Surgeon. Salary £100, with board, lodging, and washing. Appointment for six months, subject to re-election. Applications to the Secretary before December 14th.

Bath Royal United Hospital.—Resident Medical Officer. Appointment for three years. Salary £100 per annum, with board, lodging, and washing. Applications to the Secretary-Superintendent by December 17th.

Derbyshire Royal Infirmary.—Resident House Surgeon and Resident House Physician; doubly qualified. Appointments tenable for twelve months, with a possibility of extension. Salaries, £100 and £80 per annum respectively, with apartments and board. Applications, endorsed "House Surgeon" or "House Physician," to Walter G. Carnt, Secretary, by December 21st.

Swansea General Hospital.—House Surgeon. Salary £50 per annum, with board, residence, washing, and attendance. Applications to Jno. W. Morris, Secretary, 9, Castle Street, Swansea, by December 16th.

West London Hospital, Hammersmith Road, W.—House Physician and House Surgeon. Appointments for six months. Board and lodging provided. Applications to R. J. Gilbert, Secretary-Superintendent, by December 18th

##### Non-Resident Medical Officers.

Dental Hospital for London, Leicester Square, W.C.—Assistant Dental Surgeon; must be L.D.S. Applications to J. Francis Pink, Secretary, by January 6th.

Dental Hospital for London and London School of Dental Surgery, Leicester Square, W.C. — Demonstrator. Honorarium £50 per annum. Applications to J. Francis Pink, Secretary, by January 6th.

Evelina Hospital for Sick Children, Southwark, S.E.—Four qualified Clinical Assistants and eight unqualified Clinical Clerks in the Out-patient Department. Applications to the Secretary by December 17th.

Price 2s. 6d.

THE SCIENTIFIC PRESS (Limited), 428, Strand, W.C.

### BIRMINGHAM GENERAL HOSPITAL.

FOUNDED 1772.

### SUPPORTED BY VOLUNTARY CONTRIBUTIONS.

**THE ANNUAL EXPENDITURE EXCEEDS** } £16,500

**INCOME FROM INVESTMENTS** £4,000

**ANNUAL SUBSCRIPTIONS** ... £5,000

**270 Beds. Daily average occupied, 232.**

During the year 1894 there were treated 4,336 In-patients and 49,835 Out-patients (a total of 54,171 cases), of which 4,011 In and 33,630 Out Patients were admitted **without ticket.**

A NEW BUILDING is now being erected to contain 340 Beds. FUNDS ARE URGENTLY NEEDED FOR ITS COMPLETION.

**The Jaffray Branch at Gravelly Hill** (56 Beds) receives the more chronic cases from the Wards, and so enables a larger number to be admitted to the parent Institution.

**Separate Subscription List of £670.** | **Expenditure over £3,000.**

**NEW AND INCREASED ANNUAL SUBSCRIPTIONS ARE URGENTLY NEEDED,**

and will be thankfully received by

HOWARD J. COLLINS, House Governor.

### ROYAL LONDON OPHTHALMIC HOSPITAL,

### MOORFIELDS.

### FUNDS MUCH NEEDED.

| | | | |
|---|---|---|---|
| **Total Ordinary Income...** | | £3,764 | 17 6 |
| **Total Ordinary Expenditure...** | | 6,983 | 8 10 |
| **Patients** | 26,492 | | |
| **Attendances** | 113,555 | | |

ANNUAL SUBSCRIPTIONS and DONATIONS earnestly solicited.

Bankers: Messrs. Williams, Deacon, and Co., 20, Birchin Lane, E.C.

ROBERT J. NEWSTEAD, Secretary.

### NATIONAL ORTHOPÆDIC HOSPITAL,

*Now Ready. 8th Edition. Greatly enlarged and thoroughly revised. Crown 8vo. cloth gilt, 3s. 6d.*

### NURSING: ITS THEORY AND PRACTICE,

BEING A

*COMPLETE TEXT-BOOK OF MEDICAL, SURGICAL, AND MONTHLY NURSING.*

By PERCY G. LEWIS, M.D., M.R.C.S., L.S.A., &c., &c.

To this edition have been added three chapters on Monthly Nursing, together with additions to the chapters on Blood Diseases, on Antiseptics, on the Nursing of Children, and on Private Nursing. Under these headings will be found a short account of the new Aseptic Treatment, the Antitoxin and Serum Treatments, and the Treatment by Thyroid Extracts.

London: THE SCIENTIFIC PRESS (LIMITED), 428, STRAND, W.C.

### "THE HOSPITAL"

AN INDEPENDENT JOURNAL OF

**The Medical Sciences**

AND

**Hospital Administration,**

WITH WHICH IS ISSUED WEEKLY

**A SPECIAL**

### SUPPLEMENT FOR NURSES.

PUBLISHED EVERY SATURDAY.

PRICE TWOPENCE.

Matrons, Sisters, or Nurses will find in The Hospital a chronicle of the latest Nursing News, Practical Articles of the highest value to those who wish to keep themselves abreast with the times, and brightly written articles from Nurses all over the world.

**SUBSCRIPTIONS CAN COMMENCE AT ANY TIME.**

### TERMS OF SUBSCRIPTION.

### WEEKLY ISSUE.

| | Great Britain. | Foreign and Colonial. |
|---|---|---|
| For One Year ... ... | 10s. 6d. ... | 15s. 2d. |
| For Six Months ... | 5s. 3d. ... | 7s. 7d. |
| For Three Months... | 2s. 8d. ... | 3s. 10d. |

### MONTHLY PARTS.

| | Post Free to any Part of the World. |
|---|---|
| For One Year ... ... | 8s. 6d. |
| For Six Months ... ... | 4s. 3d. |
| For Three Months ... ... | 2s. 2d. |

Postal Orders and Cheques should be made payable to "The Scientific Press, Limited."

N.B.—In order to prevent delay, all business communications should be addressed to "The Manager" only, and not to any person individually.

### CHANGE OF ADDRESS.

In event of change of address, notice should be forwarded to the Publishers by the Saturday previous to date of publication, and the old address should also be given.

"The Hospital" can be obtained of all Booksellers and Newsagents in the United Kingdom, and at all the Railway Stalls, of Messrs. W. H. Smith and Son; and also from the following Agents in the Colonies and Abroad:—

Paris: Librairie Galignani, 224, Rue de Rivoli.
Berlin: Messrs. A. Asher and Co.
Leipsig: Mr. F. A. Brockhaus.
New York: The International News Co.
Montreal: The Montreal News Co.
Toronto: The Toronto News Co.
Melbourne, Sydney, Adelaide, and New Zealand: Messrs. Gordon and Gotch.
India: At all the Railway Bookstalls and Agencies of Messrs. A. H. Wheeler and Co.
South Africa: Messrs. Juta, Cape Town.

Publishing Offices: 428, Strand, London, W.C.

*In the Press. Price 5s.*

### BURDETT'S OFFICIAL NURSING DIRECTORY

A DIRECTORY, NOT A REGISTER.

**The above work may be subscribed for at a reduced price by those whose names appear in the book, and who supply full particulars on a form which can be obtained upon application to the Publishers,**

THE SCIENTIFIC PRESS (Lim.), 428, Strand, London, W.C.

Just Published. With numerous Illustrations, demy 8vo., blue buckram, gilt, 3s. 6d.

### DIET IN SICKNESS AND IN HEALTH.

**By Mrs. ERNEST HART,**

*Formerly Student of the Faculty of Medicine of Paris, and of the London School of Medicine for Women*

With an Introduction by Sir HENRY THOMPSON, F.R.C.S., M.B. Lond.

"Packed with valuable hints and practical counsel; and as human happiness is, after all, very much a matter of diet, the little volume should do much to mitigate the miseries and add to the joys of existence."—*St. James's Gazette.*

"Does not ask the reader blandly to accept such and such a diet for such and such complaint, but explains to him, briefly and simply, the why and wherefore, and gives in each case excellent physiological lessons, illustrated with comprehensive diagrams. Another good point is its cheerful, sensible tone. The book is excellent in every way, and may be warmly recommended to the enormous number of people who, for one reason or another, have to be careful about their diet."—*Westminster Gazette.*

LONDON: THE SCIENTIFIC PRESS (LIMITED), 428, STRAND, W.C.

### STANDARD TEXT-BOOKS FOR NURSES.

New and Revised Edition. Demy 8vo., 209 pp., profusely illustrated with Copyright Portraits and Drawings. Price 2s. 6d., post free.

HOW TO BECOME A NURSE: And How to Succeed. A complete guide to the Nursing Profession for those who wish to become Nurses, and a useful book of Reference for Nurses who have completed their training and seek employment. Compiled by HONNOR MORTEN (Author of "Sketches of Hospital Life," "The Nurse's Dictionary," &c.)

"To those who are frequently appealed to by girls or young women, as to the steps they should take to become nurses, this book of Miss Morten's must prove a perfect godsend."—*British Medical Journal.*

THE NURSES' DICTIONARY OF MEDICAL TERMS AND NURSING TREATMENT. By HONNOR MORTEN. 2nd Edition. Demy 16mo. Cloth 2s., leather 2s. 6d.

NURSING. By ISABEL ADAMS HAMPTON. 2nd Edition. Crown 8vo. Cloth, 500 pp. 7s. 6d. net.

ART OF MASSAGE. By A. CREIGHTON HALE. Demy 8vo. Cloth. Illustrated. 6s.

SURGICAL WARD-WORK AND NURSING. By ALEXANDER MILES, M.D. (Edin.), C.M., F.R.C.S.E. Demy 8vo. Cloth. With numerous illustrations. 3s. 6d.

HOSPITAL SISTERS AND THEIR DUTIES. By EVA C. E. LUCKES. 3rd Edition. Crown 8vo. Cloth. 2s. 6d.

ART OF FEEDING THE INVALID. By a Medical Practitioner and a Lady Professor of Cookery. Demy 8vo. Cloth, 260 pp. 3s. 6d.

London: The Scientific Press (Ltd.), 428, Strand, W.C.

